# Modelling Hurricane Exposure and Wind Speed on a Mesoclimate Scale: A Case Study from Cusuco NP, Honduras

**DOI:** 10.1371/journal.pone.0091306

**Published:** 2014-03-10

**Authors:** Sven P. Batke, Merlijn Jocque, Daniel L. Kelly

**Affiliations:** 1 Department of Botany and Trinity Centre for Biodiversity Research, Trinity College Dublin, Dublin, Ireland; 2 Koninklijk Belgisch Instituut voor Natuurwetenschappen, Brussels, Belgium; 3 Operation Wallacea, Old Bolingbroke, United Kingdom; 4 Department of Biological Sciences, Rutgers University, Newark, New Jersey, United States of America; University of Oxford, United Kingdom

## Abstract

High energy weather events are often expected to play a substantial role in biotic community dynamics and large scale diversity patterns but their contribution is hard to prove. Currently, observations are limited to the documentation of accidental records after the passing of such events. A more comprehensive approach is synthesising weather events in a location over a long time period, ideally at a high spatial resolution and on a large geographic scale. We provide a detailed overview on how to generate hurricane exposure data at a meso-climate level for a specific region. As a case study we modelled landscape hurricane exposure in Cusuco National Park (CNP), Honduras with a resolution of 50 m×50 m patches. We calculated actual hurricane exposure vulnerability site scores (EVVS) through the combination of a wind pressure model, an exposure model that can incorporate simple wind dynamics within a 3-dimensional landscape and the integration of historical hurricanes data. The EVSS was calculated as a weighted function of sites exposure, hurricane frequency and maximum wind velocity. Eleven hurricanes were found to have affected CNP between 1995 and 2010. The highest EVSS’s were predicted to be on South and South-East facing sites of the park. Ground validation demonstrated that the South-solution (i.e. the South wind inflow direction) explained most of the observed tree damage (90% of the observed tree damage in the field). Incorporating historical data to the model to calculate actual hurricane exposure values, instead of potential exposure values, increased the model fit by 50%.

## Introduction

The contribution of high energy weather events to community dynamics and large scale diversity patterns both through alterations of the community structure as well as long distance dispersal is often assumed but hard to prove [1]. The only information currently available is from studies comparing community structure and diversity before and after the passing of hurricanes. In most cases observations are collected after the unexpected passing of hurricanes [2], often through long term observation plots [3]. Such occasional observations have limited general applicability. A more comprehensive approach is synthesising weather events in a location over a long time period, ideally at a high spatial resolution and on a large geographic scale [4]. This would improve our understanding on how large weather events contribute to community dynamics and diversity patterns. Little such information is available (however see [5]), but the availability of advanced geographical software, the wealth of detailed weather data and high resolution images of geographic topography provide the means to generate such data [6].

Hurricanes are well defined and relatively predictable weather events, with a well monitored path trajectory. Hurricane storms are dynamic weather fronts that change in size, speed and intensity throughout their life time. These highly organised systems often originate over tropical and subtropical waters, moving westwards with a counter clockwise rotation in the Northern Hemisphere. The compilation of a hurricane exposure map for a particular region, expressing the accumulated impact of past hurricanes, can be calculated through the combination of 1) a wind pressure model, 2) an exposure model that can incorporate simple wind dynamics within a 3-Dimensional landscape and 3) the integration of previous hurricanes during the considered time period.

The wind pressure model is the core of the calculations and predicts how wind speed decays from the centre of the hurricane. In the past 30–40 years significant advances in sensing and analytical hurricane hind- and forecasting technology have allowed a more accurate representation and assessment of hurricane wind models and damage [7]. Of particular interest was the mathematical representation of the empirical wind-pressure relationship [8]–[10]. The relationship between minimum central pressure and maximum surface winds can be used as a basic parametric radial wind profile model, to estimate the wind impact beyond a certain radius from the hurricane centre [11]. The wind pressure model generates wind speeds for all points in a region for a passing hurricane.

The actual impact of the winds in those locations also depends on the topography of the landscape. A landscape can be broken down into localities differently affected by passing large weather events - largely determining the local mesoclimate. Topographic exposure has been defined as a geomorphometric feature that is characterised by its degree of protection by the surrounding landscape [12], [13]. The geomorphometry of a landscape can be assessed by means of digital elevation models (DEM) in the GIS (Geographical Information System) environment. The level of topographic exposure is often very difficult to model, as wind direction and velocity can change as a function of topographic complexity, vegetation type and local abiotic climate conditions [14]. A basic topographic exposure model (EXPOS) can be used that assesses wind exposure as a simple function of relative height and distance to the surrounding horizon [5], [12]. EXPOS evaluates each point on the DEM as more or less protected or exposed, providing the points fall within the wind shadow cast by points upwind. In other words, the bending inflection angle of the wind is fixed as it passes over the landscape. The degree of inflection angle can also help to categorise areas that are more or less likely to be affected by winds [5].

These two core components generate an exposure map, expressing the effective impact of a single hurricane passing through a region. Therefore, integrating individual hurricane exposure scores into a single map, will give a more relevant ecological and biogeographical image of the affected region. The practical application of EXPOS, combined with exposure models such as TOPEX (Topographic Exposure) and HURRECON (Hurricane Reconstruction) has most frequently been explored in the forestry sector [5], [15], [16], where wind damage assessments are a common tool in timber and conservation management. For example, wind damage can be a driving force in landscape level community patterns by affecting vegetation dynamics [17], when storm forces exceed the resistance of trees to either breakage or overturning [18], [19]. Applications of these models and risk assessments are commonly performed at single sites and until now direct comparisons of large and infrequent disturbances between sites are rare. This is largely due to the difficult to quantify effect of topography amongst sites [1]. An integrative model that allows for a high resolution comparison of cumulative site vulnerability over large areas would therefore be most valuable in forest management, as it can help to assess individual ecosystem (e.g. tree, stand level) responses to different exposure levels over time [4].

Additionally, geographical site information on cumulative hurricane impacts would contribute significantly to the biogeography of diversity. This will be important as a good understanding of the influence of large storms on terrestrial ecosystems and diversity patterns will grow in importance with changing climates. For example, recent modelling of tropical storm incidences shows that over the last 30 years the frequency of tropical storms remained largely the same but that intensity increased [20], [21]. Such an increased intensity of extreme weather events could reinforce the interaction between climate and community dynamics and could further influence the distribution of organisms and diversity patterns [22].

To contribute to the understanding of community structure, dynamics and diversity patterns in relation to high energy weather events, we compose a cumulative hurricane exposure map, synthesising the effect of hurricanes in the last 15 years, on a high spatial resolution (50×50 m) for Cusuco National Park in Honduras. We therefore adjusted an existing model [5], [16] to predict the impact of hurricane winds on a mesoclimate level in a landscape. Our new model adds a cumulative impact factor of historical hurricanes. The aimed outputs of the model were i) prevailing wind directions, ii) the wind speeds of historical hurricanes in a specific area and iii) a high resolution exposure map highlighting past hurricane impact.

To evaluate the model we correlated model predictions with field observations of tree damage and an onsite evaluation of topographic exposure in Cusuco National Park. Physical tree damage is almost exclusively the result of high intensity weather [23] and should be a good indicator of hurricane exposure.

## Materials and Methods

### Study Area

**Figure 1:**
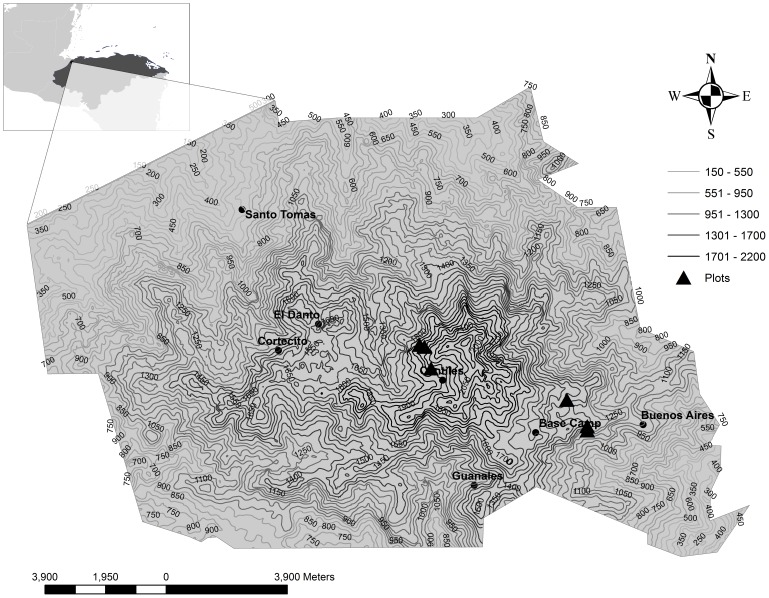
Cusuco National Park. The delineation of the Cusuco National Park (CNP) boundary and situation in Honduras (15° 32′ 31″ N and 88° 15′ 49″ W). Camp sites are illustrated on a 50 m resolution contour map, as well as plot locations of the 2012 summer season.

Cusuco National Park (CNP), at 15° 32′ 31″ N and 88° 15′ 49″ W is located in the Departmentos Santa Barbara and Cortes in NW Honduras in close proximity to the border with Guatemala (Figure 1). The park was established in 1959 and is currently designated as a National Park under Decr. 87–87 and the IUCN category II. Cusuco’s vegetation consists of semi-arid and moist tropical mountain pine forest dominated by *Pinus maximinoi* H.E. Moore and *Pinus tecunumanii* F. Schwerdtf. ex Eguiluz & J.P. Perry, and moist broadleaved forest, with dwarf forest at higher altitudes [24]. The topography of CNP is mountainous with a minimum elevation of 600 m and a maximum elevation of 2245 m within the core-zone boundary. The camp has one visitor centre (Base Camp; 15° 29′ 15″ N and 88° 12′ 42″ W), the villages St. Tomas (15° 33′ 43″ N and 88° 18′ 01″ W) and Buenos Aires (15° 29′ 59″ N and 88° 10′ 45″ W) and four camping sites (Cantiles; 15° 30′ 45″ N and 88° 14′ 22″ W, Cortecito; 15° 31′ 15″ N and 88° 17′ 20″ W, Danto; 15°31′ 43″ N and 88° 16′ 36″ W and Guanales; 15° 28′ 55″ N and 88° 13′ 48″ W).

### Modelling Hurricane Wind Fields

For this study hurricane Best Track Data (HURDAT) were obtained from the National Oceanic and Atmospheric Administration (NOAA) [25]. The data file contained hurricane track data from 1851 to 2010. We used hurricanes that intersected within a 480 km buffer from CNP and were recorded between 1995 and 2010. The 480 km buffer was chosen because it averages a typical hurricane width [26]. However, track data for stronger hurricanes (Category >3) outside the buffer were independently assessed for their potential inclusion under larger hurricane radii assumptions (i.e. >480 km). We used hurricanes from the last 15 years as data on the 6-hourly centre location (latitude and longitude) and intensities (maximum surface wind speeds and minimal central pressure) were available for these. The x/y-track data were plotted using ArcGIS 10 [27].

The model presented here to calculate the hurricane wind field was based on a basic pressure-wind model as often used in hurricane studies [7], [16]:
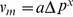
(1)where *a* and *x* are empirical constants, *v_m_* is the maximum wind speed, 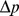 is the pressure drop from a defined external pressure to the central pressure (*p_n_ – p_c_*). In this study the external pressure was defined as *p_n_* = 1015 for the western North Pacific and *p_n_* = 1010 for the North Atlantic [9]. The constant *x* has often a typical value of approximately 0.7 for near surface winds [28] and was computed as follows [9]:



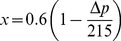
(2)Although *V_m_* was available from the data set for each hurricane, *V_m_* was recalculated by the authors using an adjusted pressure-wind model. This was done to test model performance. The model took the following equation [9]:
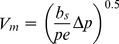
(3)where *p* is air density, *e* the base of the natural logarithm and *b_s_* is a function of the hurricane eye latitude and current pressure deficit, calculated as follows:
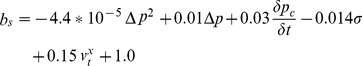
(4)where *p_c_* is the observed central pressure and 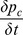 is the intensity change over time (hPa h^−1^).

**Figure 2:**
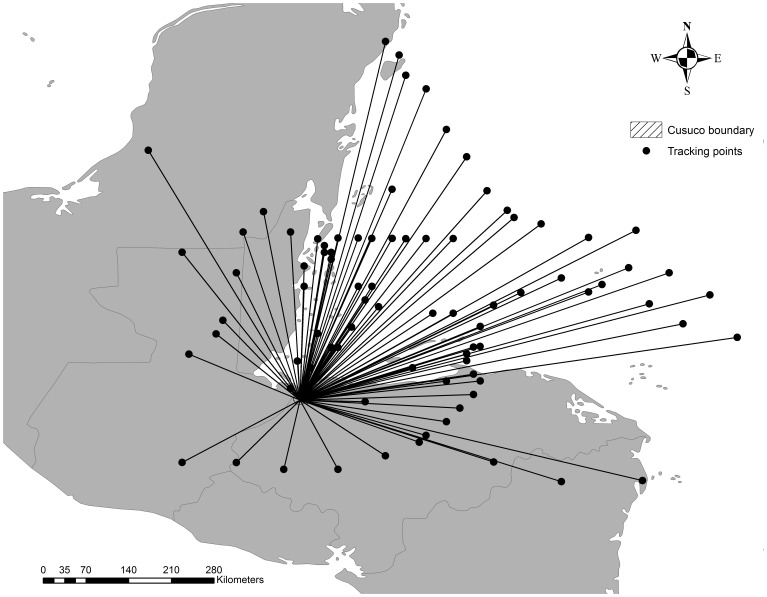
Hurricane distance calculations. The distance from every selected tracking point to the centre of CNP was calculated using the ArcGIS extension ‘Spider’.

To describe the wind speed intensity and the size of the hurricane, wind speed *V_m_* (at the eye-wall) and the radii of maximum winds *R_max_* were computed [7], [9], [29]. In order to calculate a wind gradient from the hurricane centre to the peripheral zone of each hurricane, the hurricane eye-diameter (*ED*) was calculated as:
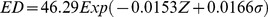
(5)where 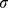 is the absolute value of latitude and 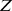 is the observed wind speed. The eye-diameter was then used to calculate the radial tangential wind speed beyond *R_max_*, *V_r_* as follows:
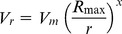
(6)where *r* is the distance and *R_max_* is the radius of maximum wind speed calculated as follows:



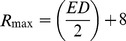
(7)The wind gradient was calculated around the hurricane centre in 15 km intervals (i.e. 15, 30, 45,… km). In ArcGIS multiple ring buffers were created using the buffer wizard tool. The buffers were based on the wind strength attributes and the fixed distance intervals. In the next step, all hurricane buffers that intersected with the boundary of CNP were extracted to identify hurricane tracking positions of the intersecting hurricanes. The selected tracking positions were finally used to calculate wind velocity based on the specific distances from each tracking point (that is the hurricane centre) to the centre of CNP (Figure 2).

Wind direction for each hurricane was calculated by finding the interior spherical angle between the hurricane centre and CNP, multiplied by the radius of the Earth as follows:
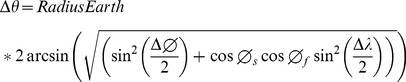
(8)where 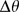 is the interior spherical angle, *RadiusEarth* was approximately 6370.97 nkm, 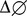 is *latitude1– latitude2*, 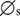 is *latitude1*, 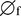 is *latitude2* and 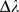 is *longitude1– longitude2*. By assuming a simplified 25 degree tangential inflow angle for hurricanes [5], [25] the wind direction was estimated in degrees for every hurricane.

The adjusted pressure-wind model (equation 5) did not perform as expected. The maximum wind was underestimated by approximately 46%, following comparison with the National Oceanic and Atmospheric Administration (NOAA) data base. As a result, wind velocity at CNP was recalculated using the NOAA *V_max_* values instead.

### Hurricane Exposure Vulnerability Site Score (EVSS)

A digital elevation model (DEM) of CNP was created using ArcGIS 10 and ArcScene 10 [27]. The DEM was based on a 50 m contour-profile map provided by Operation Wallacea (http://opwall.com/) in 2011. DEM’s represent a digital and continuous raster surface image of a terrain that can be used to visualize and perform spatial analytical model processes. The DEM was derived using the Topo To Raster tool in ArcGIS. In addition, topographic exposure was modelled using the shade topography tool (hill shade) [12]. The tool parameters were set individually for the horizontal angle to eight cardinal points (N, NE, E, SE, S, SW, W and NW) and a vertical angle of 5°, 20° and 45°. The three different inflow angles were chosen because they are most likely to reflect the upper (45°), lower (5°) and observed (20°) extremes for hurricane winds [5].

Based on the results from the DEM and the hurricane modelling, an exposure vulnerability site score (EVSS) was designated to areas based on a) the exposure score, b) hurricane frequency and c) wind velocity. The total EVSS was calculated as follows:
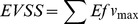
(9)


In this formula *E* is the exposure score (i.e. the weighted number of exposed and unexposed patches from the GIS raster fields) classed into five discrete categories: 1 = <45, 2 = 46–90, 3 = 91–135, 4 = 136–180 and 5 = 181–225, *f* the hurricane frequency over 15 years (1995–2010) and *v_max_* the maximum wind velocity calculated for each hurricane at CNP. The value *v_max_* was classed into three categories following the Saffir-Hurricane index (1 kt = 0.514 m/s) that is 1 = <20–34 kt (tropical depression), 2 = 35–63 kt (tropical storm) and 3 = >63 kt (hurricane). The final EVSS’s for each cardinal aspect was the weighted sum of the exposure score, the hurricane frequency and the maximum wind velocity. The final EVSS’s score were then used as an indicator score for cumulative historical hurricane damage.

### Model Evaluation

To test how well the exposure model performed, the scores derived from the DEM (that is EVSS) were validated on the ground using an adapted Climatological Observer’s Link (COL) and visual tree assessments [30]. COL is based on the observer’s subjective interpretation of the degree of shelter attributed to a location (scores 0–9). The COL scores (observed EVSS = oEVSS) were correlated with physical tree damage observed within the plots. Damage within each tree was assessed using rope access methods. Each branch on the tree was assessed for signs of storm damage and an overall damage score was computed based on signs of uprooting, bending or breakage. The oEVSS and branch damage scores were than compared to the model derived EVSS’s using regression analysis. Two models were tested. In model one the oEVSS and the observed branch damage was validated against the EVSS model results. Model two, on the other hand, used the model exposure scores only. This was done to test the fit between the simple (exposure only) and more complex (EVSS) model.

Between June and August 2012 a total of six plots were sampled (Figure 1). Plots consisted of 150 m×150 m quadrates. The locations of the plots were selected randomly within the predictive categories from the EVSS maps and a contour map of CNP. Elevation of the plots within each site was kept between 1300 and 1900 metres. The minimum distance between plots was 50 m, which was slightly lower than for other forest studies [31]. Plots were selected to compare high (EVSS 4–5) and low (EVSS 1–2) exposure sites for model validation at two locations, namely Base Camp (BC) and Cantiles (CA). At BC two high exposure and one low exposure site were selected, whereas at Cantiles two low exposure and one high exposure site were chosen. Within these model restrictions, plots were selected based on the presence of two tree types (broadleaved and *Pinus* sp.) to facilitate comparison of physical damage to trees between plots.

The model was developed using Microsoft Excel 2010 [32], ArcGIS 10 [27] and R (R Development Core Team 2012 version 2.14.0 [33]). Significance testing between different EVSS’s and model performance was assessed using Analysis of Variance (ANOVA) and Linear Modelling (LM).

## Results

**Figure 3:**
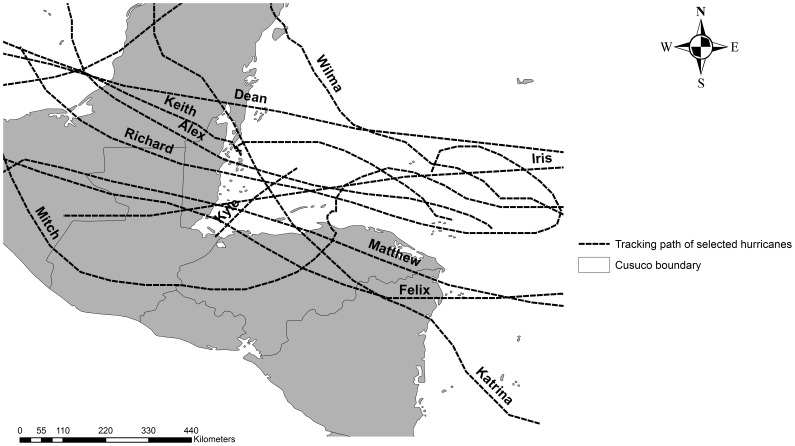
Hurricane pathways. The eleven hurricane pathways that were used to calculate the EVSS’s for CNP.

**Figure 4:**
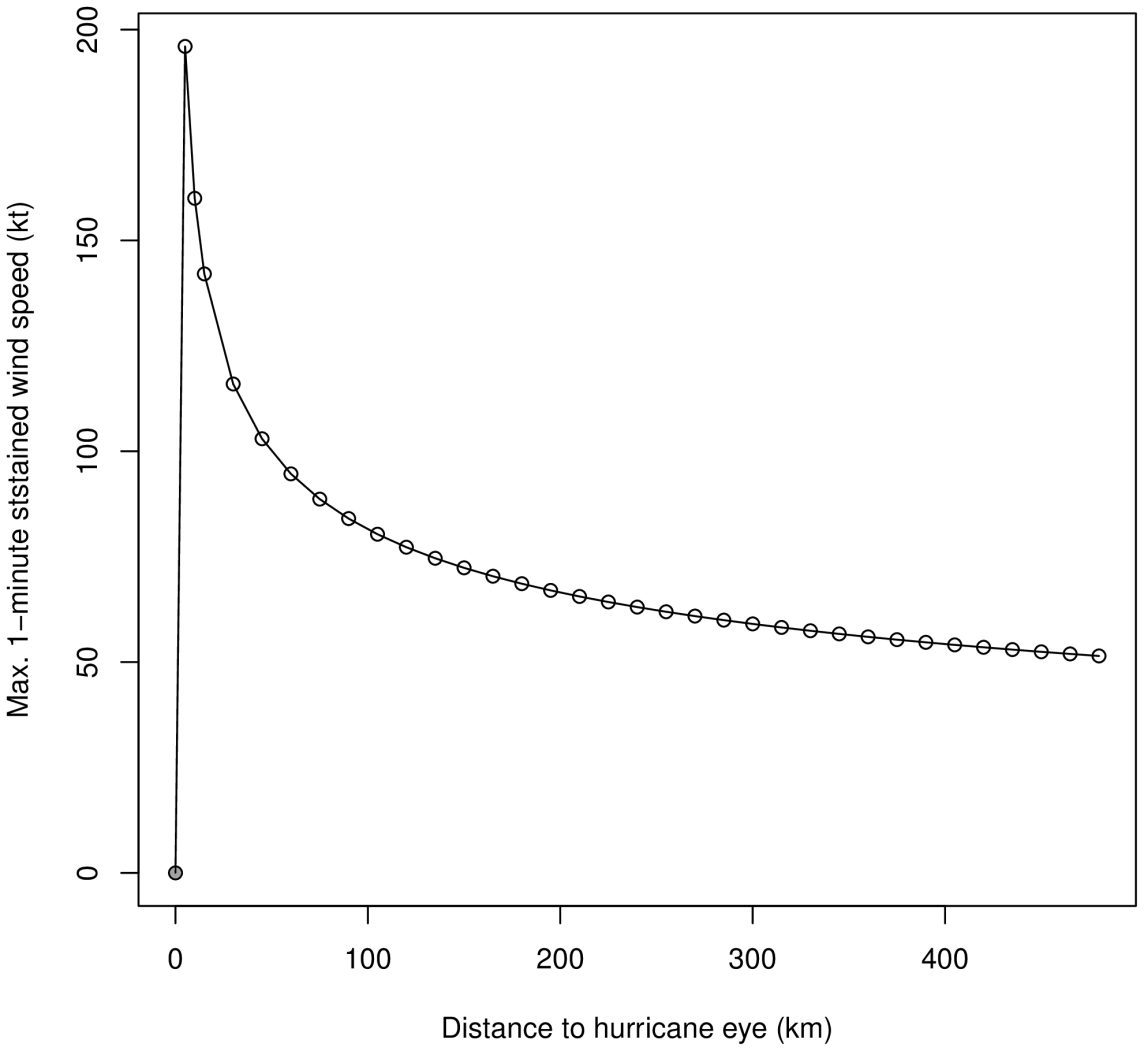
Hurricane ‘Mitch’ vortex shape. Example of vortex shape of hurricane gradient wind field, modelled for Hurricane Mitch (1998).

Of the 27 hurricanes that were selected only eleven hurricane wind fields impacted CNP and were included in the modeling (Table 1; Figure 3). Moreover, the vortex-shape of the hurricane wind field changed with each hurricane, as calculated by the model. For example, hurricane ‘Mitch’ (1998) was one of the strongest hurricanes observed, with a maximum sustained wind speed of 156.42 kt and an estimated wind speed of approximately 55 kt at 318.47 km from the ‘eye’ (Figure 4). In addition, as suggested by the wind-pressure model, wind speed was negatively correlated with air pressure (R^2^ = 0.86, P<0.001).

**Table 1:** Hurricane wind speeds.

Names	Mean sustained winds (kt)	Mean distance to Cusuco (km)	Mean(min./max.) winds at Cusuco (kt)
Alex	56.48	241.99	15.17(14.02/15.77)
Dean	126.00	474.15	37.45(17.29/53.02)
Felix	52.76	266.37	15.11(7.92/32.53)
Iris	106.45	268.29	31.55(16.7/50.47)
Katrina	26.07	196.46	8.60(8.31/8.88)
Keith	77.90	284.02	21.25(8.12/35.82)
Kyle	35.63	104.05	18.68(13.20/28.29)
Matthew	36.21	172.34	12.91(10.65/15.88)
Mitch	89.92	318.47	26.08(9.40/48.85)
Richard	68.65	233.73	18.84(9.91/25.77)
Wilma	132.02	539.82	41.66(26.49/63.50)
**Total**	**808.08**	**3099.7**	**247.28(142.01/378.78)**
**Mean**	**73.46**	**281.79**	**22.48(12.91/34.43)**
**StD.**	**36.57**	**126.53**	**10.53(5.61/17.66)**

The mean sustained hurricane wind speed in knots (kt) as taken from NOAA [25], mean distances in kilometre (km) from selected tracking-points to CNP and the modelled mean, minimum and maximum wind speeds at CNP are listed.

Most hurricane winds that struck CNP were relatively weak with a mean wind speed of 22.35 kt; hurricanes after landfall showed a strong reduction in strength with distance from the centre. The strongest hurricane was ‘Wilma’ (2005) with a cumulative mean sustained wind speed of 132.02 kt and a maximum sustained wind of 63.5 kt at CNP. On the other hand hurricane Katrina (1999) had only a cumulative mean sustained wind speed of 26.07 kt and a maximum sustained wind of 8.88 kt at CNP (Table 1).

Wind direction, assuming a 25° inflow angle, changed between hurricanes and hourly hurricane eye positions (Table 2). South and South-East winds were most commonly observed, whereas north winds rarely occurred. The most northerly winds at CNP came from hurricane ‘Mitch’ (1998), which circled CNP from the South, then along the West site of CNP, then to the North. On the other hand, hurricane ‘Wilma’ (2005), for example, varied less in wind direction (Table 2).

**Table 2:** Hurricane wind directions.

	Mean (min./max.) wind direction (°)				
Names	0 h	6 h	12 h	18 h	Mean
Alex	151.0(−/−)	96.8(−/−)		196.2.1(−/−)	**148.0**(96.8/196.2)
Dean	165.5(−/−)	175.0(−/−)		109.2(72.4/146.0)	**139.7**(72.4/175.0)
Felix	192.3(112.3/272.2)	287.5(−/−)	248.7(241.0/256.4)	191.9(126.5/257.3)	**221.9**(112.3/287.5)
Iris	209.9(−/−)	109.7(−/−)	160.4(−/−)	187.9(−/−)	**167.0**(109.7/209.9)
Katrina	200.4(−/−)	197.6(−/−)			**199.0**(197.6/200.4)
Keith	173.7(144.9/191.1)	164.3(98.6/193.8)	163.8(193.5/192.4)	188.5(183.7/192.7)	**171.3**(93.5/193.8)
Kyle	205.9(−/−)	211.8(−/−)	217.7(−/−)	165.4(131.8/199.0)	**193.2**(131.8/217.7)
Matthew		197.1(−/−)	211.1(−/−)	111.4(−/−)	**172.2**(111.4/211.1)
Mitch	179.0(16.8/293.4)	208.5(132.6/298.1)	163.7(29.7/284.0)	169.5(26.0/288.4)	**180.9**(16.8/298.1)
Richard	155.3(−/−)	104.4(−/−)	143.1(−/−)	199.4(−/−)	**149.1**(94.7/199.4)
Wilma	154.9(147.5/161.8)	150.7(136.5/162.0)	153.8(143.4/165.2)	155.8(150.8/165.0)	**153.6**(136.5/165.2)

Mean wind direction from hourly hurricane centre location towards CNP [25].

### Exposure Vulnerability at CNP

**Figure 5:**
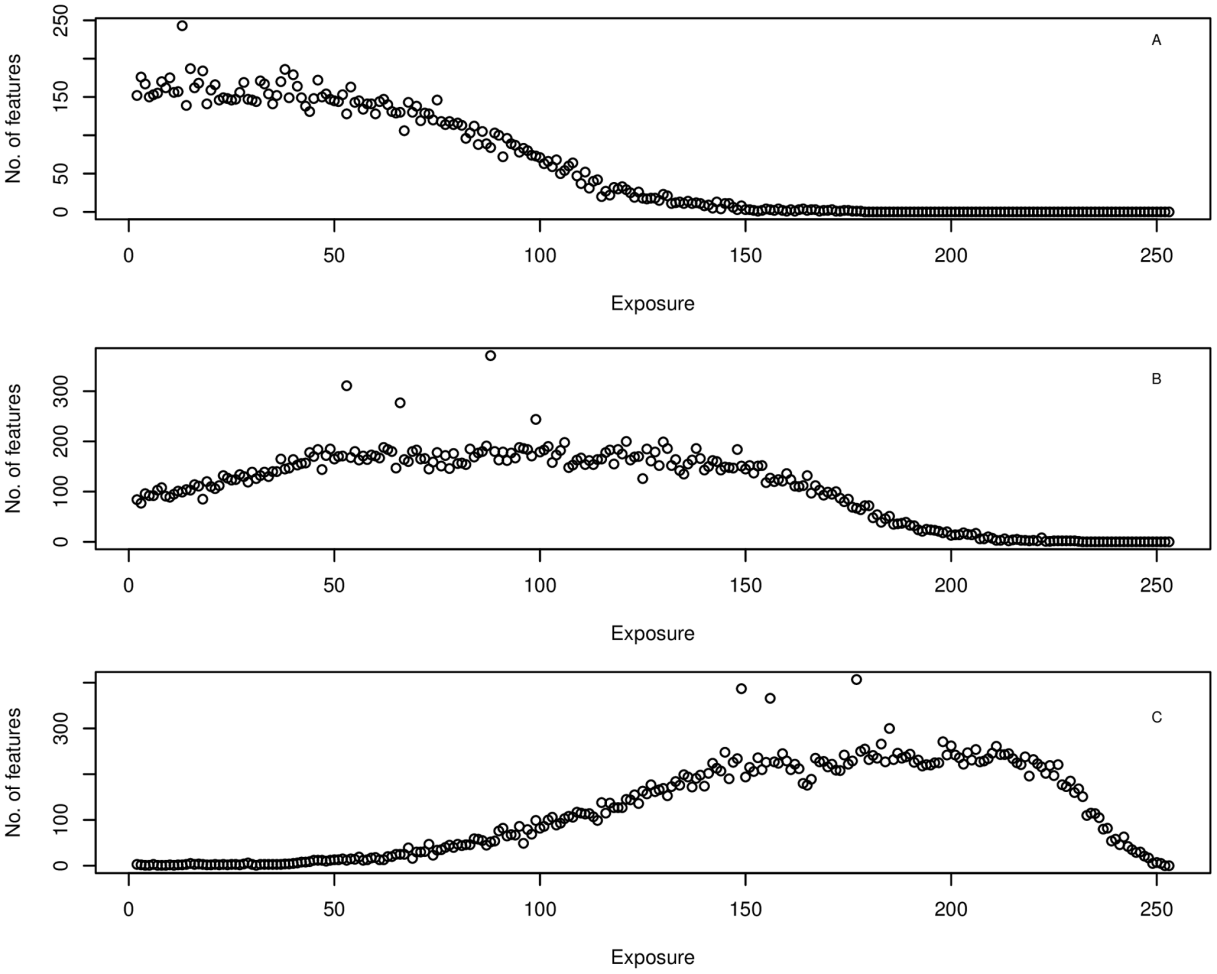
Exposure distribution curves. Exposure distributions under three different inflow angles, that is 5° (A), 20° (B) and 45° (C). An exposure value of 0 indicates sheltered conditions, whereas higher values indicated an increase in exposure. The number of different features refers to the number of 50 m×50 m squares on the DEM.

Modelling topographic exposure at CNP, using different inflection angles (5°, 20° and 45°), yielded different site-exposure distribution curves (Figure 5). At a 5° (A) inflow angle the curve took a sigmoidal distribution, with the majority of sites not being exposed. At an angle of 20° (B) and 45° (C) however, the difference of exposure between exposed and unexposed sites was more normally distributed. Therefore, site exposure increased with an increasing infliction angle. With regards to modelled hurricane wind speed and direction, the strongest maximum winds were estimated to have occurred at the South-East (63.5 kt) side of the park, followed by the South (53 kt), North-East (48.6 kt), North-West (47.9 kt), West (33.6 kt), South-West (32.5 kt), East (18.1 kt) and North (13.8 kt).

The EVSS’s were calculated using hurricane frequency, maximum wind speed and exposure scores (equation 9). The most vulnerable sites, that is with the highest EVSS’s, were bare mountain crests, whereas sheltered valley-basins had lower EVSS’s. However, EVSS’s did not directly change with altitude, but were more a combined response to protection from direct wind influence and altitude. Average EVSS’s for the whole region considered, varied significantly between cardinal directions (F_9,14_ = 1.754, P<0.001) but not between inflow angles (F_2,21_ = 0.08, P<0.05). The South and South-East facing sites at CNP had the highest EVSS’s (P<0.001), followed by the North-West and North-East facing sites (P<0.05).

**Figure 6:**
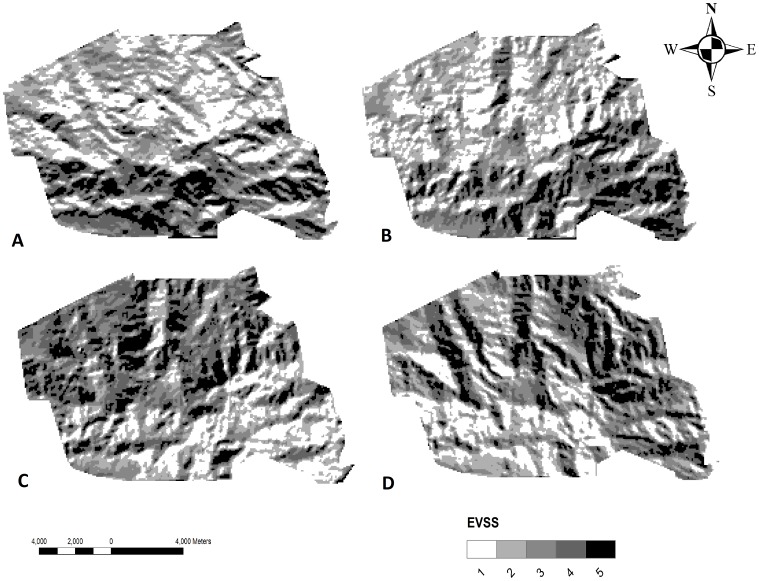
Exposure vulnerability site score maps. Exposure vulnerability site score (EVSS) maps based on equation 9, assuming an inflow angle of 20°. An EVSS of one indicates low site vulnerability and a high score high vulnerability. Note that only the wind inflow direction from the South (A), South-East (B), North-West (C) and North-East (D) are shown. EVSS’s map scores correspond to a range of approximately 28–34 (A), 23–32 (B), 5–7 (C) and 5–7 (D).

Because the different inflow angles did not affect the overall EVSS’s, the final EVSS maps were derived from a 20° inflow angle [5]. Figure 6 presents four maps (A-D) from the four different wind inflow directions that had the highest observed EVSS scores. It needs to be noted, however, that the vulnerability map scores between the different maps were adjusted to aid visualisation. For example, Figures 6A and 6B had EVSS’s in the range between 23–34, whereas Figures 6C and 6D had much lower EVSS’s, in the range between 5–7 (Figure 6).

Mostly high vulnerability was observed on CNP sites North-West from Guanales and South-East from Base-Camp and Buenos Aires (South and South-East inflow direction). On the other hand, sites with low EVSS’s under a South and South-East inflow wind direction, were to be found in the north-west of the Park, in the vicinity of Santo Tomas and Cortecito (Figure 6 A–D).

### Model Evaluation

As expected, branch damage (n = 647) on the individual trees (n = 30) had a strong positive correlation with the plot oEVSS (Table 3). Model one explained more of the variation in the data than model two. Moreover, branch damage and oEVSS were significantly correlated with the South hurricane solution but not with the South-East solution (Table 3). Other model solutions were not statistically significant following Akaike Information Criterion and likelihood analysis (P>0.05; not shown).

**Table 3:** Model comparison.

Model	Treatment	Std. Error	t value	Adj. R-squared	p-value	Probability
Branch damage ∼ oEVSS	oEVSS	0.021	4.856	0.819	0.008	**
**Model 1**						
oEVSS ∼ EVSS_South+EVSS_SouthEast	South	0.864	4.601	0.904	0.019	*
	South East	0.844	−2.830	0.904	0.066	.
Branch damage ∼ EVSS_South	South	0.048	3.676	0.715	0.021	*
Branch damage ∼ EVSS_SouthEast	South East	0.063	2.433	0.496	0.072	.
**Model 2**						
oEVSS ∼ Exposure_South+Exposure_SouthEast	South	0.001	0.206	0.400	0.838	
	South East	0.001	−3.997	0.400	0.000	***
Branch damage ∼ Exposure_South	South	<0.001	−0.490	−0.027	0.628	
Branch damage ∼ Exposure_SouthEast	South East	<0.001	−3.413	0.269	0.002	**

Best fit linear model results assessing regression fit of generated exposure vulnerability site score (EVSS) from the South and South East with observed exposure vulnerability site score (oEVSS) and branch damage in two models: purely based on topography (model 1) and topography including frequency and actual wind speed (model 2). The best statistical model was chosen using Akaike Information Criterion (AIC). Significant codes are as follows: <0.001 = ‘***’, 0.001 = ‘**’, 0.01 = ‘*’, 0.05 = ‘.’, >0.05 = ‘ ’.

## Discussion

We generated a high spatial resolution (50×50 m) hurricane exposure map for Cusuco National Park that explained up to 90% of the observed tree damage on the ground. We modeled actual hurricane exposure values, based on data from the last 15 years. Actual hurricane exposure values differ from potential hurricane exposure values [5] in that they synthesise the impact of all hurricane events over a specified period. Potential hurricane exposure values present the maximal hypothetical exposure to hurricane winds from a certain direction calculated purely on topographic parameters such as slope, orientation and elevation. We compared the model fit of both the exposure values alone and the values from the complete model (EVSS) to explain physical tree damage. Our model predicted that for exposure values alone, the South-East solution performed significantly better than the South solution (Table 3), the total solution explaining 40% (Adjusted R-squared = 0.400) of the observed variance. However, when the ground data were tested as a function of EVSS, the South solution performed significantly better (Adjusted R-squared = 0.904) (Table 3). Our results showed that the calculation of actual values, instead of exposure values only, can change the relevance of wind direction and so increase the model fit by 50%.

Only eleven hurricanes affected CNP in the last 15 years, and thus only eleven events were incorporated in our model. Moreover, many only reached tropical storm strength. Despite the low number and in many cases relatively low strength of hurricanes, the model still efficiently explained the majority of hurricane related field observations at CNP. Most probably the calculation of actual hurricane exposure values from the last 15 years allowed the identification of major hurricane pathways along the reserve. As repetitive wind damage can result in forest stand adaptations to disturbance (e.g. changes in species composition and forest structure), particularly when the frequency and intensity of the disturbances are high [34], it can be predicted that forest communities will respond along this impact gradient. The long-term recovery of forests after wind disturbance has been shown to vary greatly between stands and species and is often very slow [35]. In a review on forest damage and recovery after catastrophic winds it was pointed out that forest recovery can be divided into four components: namely regrowth, recruitment, release or repression [34]. Each component is controlled by biotic and abiotic site conditions and the frequency and severity of disturbance events. As these components vary considerably between species, repetitive wind damage will alter the recovery dynamics by impeding a fast recovery for some species and/or stands.

We found that 70% of the variation in physical tree damage was explained by our South-model solution, with the majority of branches on standing trees showing signs of breakage and bending stress. These results are similar to other studies in the Caribbean [36], which found that the main direct effects by hurricanes were defoliation, uprooting and snapping; whereas indirect effects included ultraviolet damage to understory juveniles [37], alteration in species recruitment, the forest microclimate and species composition [38]. In the case of CNP the damage inflicted on trees by severe winds was significantly affected by the exposure of the trees [39], but more importantly, by the frequency and the maximum speed of hurricane winds that occurred at these sites. Wind damages on stands can be variable [40] and often are controlled by the vegetation height, composition and specific site characteristics [4]. For example, it has been shown that damage probability increases with tree diameter and tree species. Trees such as *Pinus taeda* L. showed the highest risk probability followed by *Liquidambar styraciflua* L., *Quercus* spp. and other broadleaves [4]. Similar results were found elsewhere [35]. Given that significant stretches of forest in CNP are dominated by *Pinus* spp., this could potentially further alter forest dynamics along the hurricane impact gradient. It would therefore be desirable to include structural and floristic vegetation data of the affected region [4], [40]. However, in the case of CNP this is currently restricted by the limited account and comprehensive knowledge of the forest’s vegetation.

Our model’s mathematical representation of a hurricane was simplified, and we briefly would like to point out a couple of possible points for amelioration of the model. Firstly it was assumed that the storms were circular in section. Hurricanes, however, are dynamic weather fronts that constantly change in size, shape, direction and strength [5]. Also, the model did not account for precipitation changes or strong convective cells. Soil erosion due to high precipitation [41] could increase the overall effect hurricane winds have on the local vegetation by increasing the risk of uprooting [42]. As CNP lies within a very mountainous region (80% of the slopes in Honduras exceed 20%) [43], increased soil erosion after severe rain becomes very likely [41]. Evidence of this was seen in Honduras and Nicaragua following hurricane Mitch in 1998, when intense rain triggered widespread landslides and flooding [41]. The risk probability of landslides was strongly correlated with slope and land cover type [41]. Therefore our predicted EVSSs’ are likely to be conservative, as the inclusion of vegetation and soil erosion probability could increase the overall vulnerability to tree damage (e.g. weakening of the rooting substrate and increased mechanical stress).

In conclusion, the model used here is a powerful tool to identify major hurricane pathways in the landscape and provides the means to evaluate the historical impact of hurricanes on ecosystems, environmental stability and diversity patterns of organisms. The model provides readily interpretable data in the form of discrete hurricane exposure values, wind speed direction and intensity that can easily be applied to test hypotheses.
